# Impact of COVID-19 and mitigation plans on essential health services: institutional experience of a hospital in Ethiopia

**DOI:** 10.1186/s12913-021-07106-8

**Published:** 2021-10-16

**Authors:** Firaol Dandena, Berhanetsehay Teklewold, Dagmawi Anteneh

**Affiliations:** 1 Department of Surgery, Saint Paul Hospital Millennium Medical College, Addis Ababa, Ethiopia; 2 Saint Paul Hospital Millennium Medical College, Quality Improvement and Clinical Governance Directorate, Addis Ababa, Ethiopia

**Keywords:** Essential health services, Triple setup, COVID-19, SPHMMC, Ethiopia

## Abstract

**Background:**

Health systems around the world are being challenged by an on-going COVID-19 pandemic. The COVID-19 pandemic and associated response can have a significant downstream effect on access to routine health care services, and indirectly cause morbidity and mortality from causes other than the disease itself, especially in resource-poor countries such as Ethiopia. This study aimed to explore the impact of the pandemic on these services and measures taken to combat the effect.

**Methods:**

The study was conducted at St. Paul’s hospital millennium medical college (SPHMMC) from December 15, 2020 to January 15, 2021 using a comparative cross-sectional study design. We collected data on the number of clients getting different essential health care services from May to October 2019 (Pre COVID) and the same period in 2020 (during a COVID-19 pandemic) from the patient registry book. The analysis was done with SPSS version 24 software.

**Result:**

Overall, the essential services of SPHMMC were affected by the COVID-19 pandemic. The most affected service is inpatient admission, which showed a 73.3% (2044 to 682) reduction from the pre-COVID period and the least affected is maternal service, which only decreased by 13% (3671 to 3177). During the 6 months after the COVID-19 pandemic, there was a progressive increment in the number of clients getting essential health services.

**Conclusion and recommendation:**

The establishment of a triple setup for fighting against COVID-19, which encompasses non-COVID services, an isolation center and a COVID-19 treatment center, played a vital role in preserving essential health services.

## Introduction

The outbreak of pneumonia of an unknown ethology was reported in December 2019 in Wuhan, Hubei Province, China [1]. Following this, a novel coronavirus, coronavirus disease 2019 (COVID-19), was identified as the causative virus for the pandemic in China and other parts of the world by the World Health Organization (WHO) [2].

As of March 25, 2021, more than 235 countries/territories are affected with more than 120,383,289 cases and 2, 666, 916 deaths were recorded worldwide. In Africa, 57 countries/territories were affected by more than 4,124,997 COVID-19 cases and more than 109,586 deaths were reported across the continent. On March 13, 2020 a COVID-19 positive case was first reported in Ethiopia. As of March 25, 2021, a total of 187,365 confirmed COVID-19 cases and 2657 deaths were recorded in the country [3].

The supply and demand for health care services have been shown to affect access to these services in the past. Patients may be hesitant to seek treatment because they believe health facilities are infected, or they have doubts about the competence of health care providers to implement adequate infection prevention and control procedures. When health systems are overburdened by outbreaks, people may be unable to get the care they need, resulting in an increase in the number of deaths from COVID-19-related and non-COVID-19 causes [4–6].

Many countries are struggling to strike a balance between COVID 19-related initiatives and the necessity to continue providing other critical health services. Many routine and optional services have been halted, and new techniques are being developed in response to the changing pandemic situation [7].

A country’s ability to maintain the supply of basic health care is determined by a number of factors. These are baseline health system capacity, illness burden, and COVID-19 transmission locally [8].

The Ethiopian essential health service package includes reproductive, maternal, neonatal, child, and adolescent health services; major communicable diseases; non-communicable diseases; surgical care; and emergency and critical care [9].

The Ebola outbreak in 2014–2015 resulted in an 18% drop in average health-care utilization. The decline was even greater for maternity and children’s health services, such as facility-based deliveries, which fell by 28%. The number of deaths from measles, malaria, HIV/AIDS, and tuberculosis has increased. Which was attributable to health system failures to provide those services [10–12].

Studies done in 22 hospitals in France (a country that has been struck early and with a high intensity by the COVID-19 wave) showed a 26% decline in all emergency department visits, including a decrease of 34% in strokes, 32% in transitory ischemic attacks, 64% in unstable angina, 42% in appendicitis and 36% in seizures [13, 14].

According to Global finance faculty prediction in Ethiopia there will be 238,000 women without access to facility-based deliveries, 15% percent increase child mortality and 8% increase in maternal mortality over the next year, due to the compromise in all essential services [15].

A study done in Switzerland showed a 43% decrease in elective visceral surgical procedures was observed after Covid-19 (295 vs. 165, *p* < 0.01), while emergency operations (all specialties) decreased by 39% (1476 vs. 897, *p* < 0.01). Fifty-two and 38 major oncological surgeries were performed, respectively, representing a 27% decrease (*p* < 0.316). Outpatient consultations dropped by 59%, from 728 to 296 (*p* < 0.01) [16].

## Methods

### Study setting and design

This comparative cross-sectional study was conducted on December 15, 2020 to January 15, 2021 at St. Paul’s Hospital Millennium Medical College (SPHMMC), the second largest hospital in Ethiopia located in Addis Ababa. Established in 1969, the hospital provides different medical care services to an estimated 1,000,000 people every year [17].

### Data collection and procedure

We collected data on the number of clients attending different essential health care services from patient registry books in different service areas of the hospital from May 1st to October 31, 2019 and from May 1st to October 31, 2020. Aggregate data that was collected during the emergence of the COVID-19 pandemic was compared with the number of visits in the same six months of the previous year (pre-COVID). Data collectors also asked interview questions and observed service areas of the hospital.

### Data analysis

Data was coded, entered and cleaned using the SPSS version 24 software package by the principal investigator. Simple descriptive statistics such as frequency distribution were done as appropriate and the results were presented in tables and graphs.

## Results

Taking an average of six months from (COVID) season to (pre-COVID) season, this study analyzed the impact of the COVID-19 pandemic on six essential services provided at SPHMMC. Outpatient visits fell by 57% from pre-covid to post-covid (37,739 to 16,121) (*P* 0.0126) (Table 3). From 13,888 to 6489 (53.27%), 6431 to 3000 (53.35%), 2112 to 1006 (52.37%), and 1460 to 647 (55.68%), respectively, the average number of patients visiting medical, surgical, pediatrics, and psychiatry average outpatient visits decreased by more than half compared to the same period the previous year.

During the COVID season, the average number of patients attending emergency departments decreased by 47% (4190 to 2204) (*P* < 0.0420) (Table 3). Patient visits to pediatric and adult emergency departments decreased by 70% from pre-COVID levels (from 554 to 164) and (1526 to 485) respectively. On average, the number of patients accessing our trauma wing, Addis Ababa Burn, Emergency, and Trauma Hospital (AaBET Hospital), decreased by 45% (966 to 527) per month (Tables 1 & 2).

**Table 1:** Number of visits to essential healthcare–delivering units in SPHMMC (from May 1to October 31, 2019)

Essential healthcare type	May 2019	June 2019	July 2019	August 2019	September 2019	October 2019	Monthly average
**Outpatient services**							
Internal Medicine	12,413	15,627	13,913	15,775	12,008	13,596	13,888
Surgery	5605	6650	5780	6805	7925	5821	6431
Gynaecology	3400	3765	3199	3412	3156	2808	3620
Dermatology	1027	1217	1180	1104	1648	1432	1268
Dental & Maxillofacial	888	930	1890	1756	1596	1049	1351
Ophthalmology	7102	3651	3110	4438	4606	4713	4603
Psychiatry	1450	1289	1181	1510	1696	1637	1460
Ear Nose & Throat (ENT)	1330	1596	1867	1814	1706	1598	1651
Renal Transplant	242	216	240	223	853	195	328
Palliative care	6	5	6	5	4	4	5
Oncology	1182	1071	915	1007	1038	920	1022
Pediatrics	1749	2059	2235	2055	2188	2389	2112
**Emergency services**							
Pediatrics Emergency	503	610	499	579	549	554	554
Adult Emergency	1330	1255	1217	1348	2815	1193	1526
Gynaecology Emergency	929	1346	997	979	1462	1156	1144
AaBET Emergency	948	1018	954	997	1223	658	966
**Other services**							
Family planning	276	541	426	416	551	435	440
Cervical cancer screening	14	14	22	12	17	16	16
Voluntary Counselling & Testing	49	69	79	82	69	47	67
Post Exposure Prophylaxis	5	15	10	10	15	4	10
Dialysis	95	95	93	92	413	81	144
**Maternal Service**							
Spontaneous Vaginal Delivery	495	530	502	474	532	499	505
Instrumental delivery	41	70	65	54	73	59	60
Caesarean section	303	370	384	368	394	393	368
Maternal death	1	1	0	0	0	0	–
Antenatal care	739	1124	2531	2592	2868	2652	2084
Postnatal care	503	516	552	668	880	702	636
**Surgical Service**							
Emergency surgery	138	329	248	253	273	286	254
Elective surgery	243	446	399	393	405	423	384
**Inpatient Service**							
Surgical ward	330	370	366	336	378	349	355
Medical ward	83	91	89	92	94	55	84
Pediatrics ward	55	82	73	67	68	65	68
Maternity ward	452	464	523	515	571	456	497
Gynaecology ward	108	141	161	131	175	209	154
*Others	662	891	889	833	1183	862	886
**Total inpatient admission**	1690	2039	2101	1974	2469	1996	2044

*Others: Dental & Maxillofacial, Ophthalmology, Psychiatry, ENT, ICU and Renal Transplant

**Table 2:** Number of visits to essential healthcare–delivering units in SPHMMC (from May 1to October 31, 2020)

Essential healthcare type	May 2020	June 2020	July 2020	August 2020	September 2020	October 2020	Monthly average
**Outpatient services**							
Internal Medicine	3297	6172	6497	7010	8141	7820	6489
Surgery	2551	2255	2254	3235	3725	3976	3000
Gynaecology	769	866	890	1128	1502	1477	1105
Dermatology	0	58	186	326	475	686	289
Dental & Maxillofacial	252	169	184	242	388	711	324
Ophthalmology	232	494	928	1696	2495	3572	1569
Psychiatry	602	598	615	697	594	777	647
Ear Nose & Throat (ENT)	0	234	510	629	879	1078	555
Renal Transplant	170	270	267	259	333	350	275
Palliative care	1	0	2	3	4	4	3
Oncology	538	804	832	1016	1075	987	879
Pediatrics	473	418	917	690	2168	1373	1006
**Emergency services**							
Pediatrics Emergency	173	116	172	187	254	205	164
Adult Emergency	426	400	554	571	701	694	485
Gynaecology Emergency	1063	1002	879	1004	1135	1064	1028
AaBET Emergency	629	538	260	372	595	431	527
**Other services**							
Family planning	220	178	298	230	286	265	223
Cervical cancer screening	1	0	–	–	17	16	6
Voluntary Counselling & Testing	0	0	6	77	4	0	14
Post Exposure Prophylaxis	0	1	–		15	0	3
Dialysis	–	–	206	194	211	200	68
**Maternal Service**							
Spontaneous Vaginal Delivery	443	537	442	291	529	528	462
Instrumental delivery	45	38	33	33	47	25z	37
Caesarean section	372	347	356	356	426	399	376
Maternal death	0	0	0	1	1	0	–
Antenatal care	1860	2235	1605	1352	1119	1307	1746
Postnatal care	384	520	558	509	880	427	546
**Surgical Service**							
Emergency surgery	203	148	209	192	270	245	193
Elective surgery	142	133	181	190	206	344	168
**Inpatient Service**							
Surgical ward	**–**	**–**	130	145	230	261	128
Medical ward	**–**	**–**	76	72	81	66	49
Pediatrics ward	**–**	**–**	74	75	73	70	49
Maternity ward	**–**	**–**	491	503	607	524	354
Gynaecology ward	**–**	**–**	114	127	162	142	91
*Others	211	166	828	524	648	650	504
**Total inpatient admission**	211	166	1713	1446	1801	1713	682

*Others: Dental & Maxillofacial, Ophthalmology, Psychiatry, ENT, ICU and Renal Transplant

Family planning services decreased by 47% (440 to 233) whereas dialysis services decreased by 53% (144 to 68) during the COVID season as compared to a similar six-month period prior to the emergence of the COVID-19 pandemic.

When compared to other essential health services, maternal services were the least affected, with only a 13% decrease (3671 to 3167). The average number of mothers attending antenatal visits decreased by 15% (2084 to 1746), while postnatal visits decreased by 16%. In terms of facility birth services, spontaneous vaginal deliveries fell by 9% (505 to 462) and instrumental deliveries fell by one-third (60 to 37), whereas the number of caesarean sections performed increased somewhat during the COVID-19 pandemic (Tables 1 & 2).

The average number of emergency surgeries done during the COVID-19 pandemic showed a 24% reduction (254 to 193), whereas the average number of elective surgeries decreased by 56.3% (384 to 168) as compared to a similar six-month period in the pre-COVID year. The most affected service by the COVID-19 pandemic is inpatient admissions, which showed a 73% (2044 to 682) reduction from a similar six month period prior to the COVID-19 emergence and a prominent effect on surgical admissions, decreasing by 64% (355 to 128) (Tables 1 & 2).

**Fig. 1:**
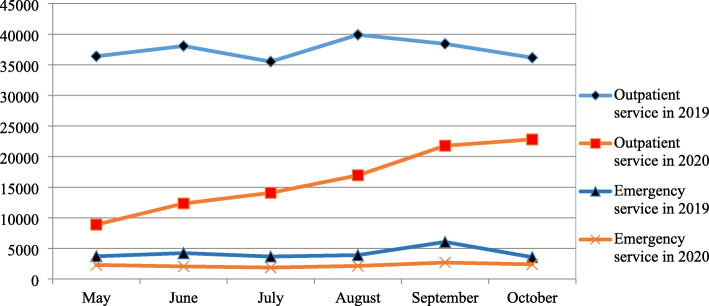
The number of client who has visited outpatient and emergency department of SPHMMC in 2019(pre-COVID 19 season) and 2020(COVID 19 season)

**Fig. 2:**
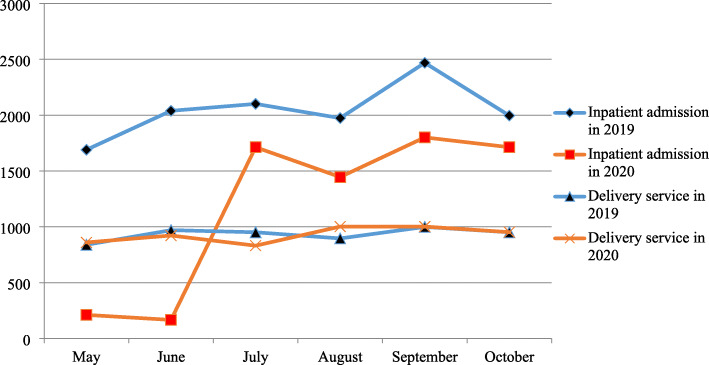
The number of client admitted to inpatient wards and number of deliveries at SPHMMC in 2019(pre-COVID 19 season) and 2020(COVID 19 season)

Regarding the number of patients visiting SPHMMC during the consecutive six months after the emergence of COVID-19 in Ethiopia, there is an overall increment. The number of patients visiting outpatient departments increased by 61% (8885 to 22,811), whereas visits to different emergency departments increased by 5% (2291 to 2394) over a period of six months. The number of surgeries (both elective & emergency) has increased by 41% (345 to 589), whereas inpatient admissions by 88% (211 to 1713). Unlike the overall increment, maternal services decreased by 13% (3104 to 2686) (Figs. 1 & 2).

## Discussion

Overall, the essential services of SPHMMC were affected by the COVID-19 pandemic. In maternity and emergency services, a higher number of clients were provided than predicted by the Ethiopian Federal Ministry of Health. This could be due to the lack of limits on maternity and emergency care, as opposed to other non-emergent services that were stopped completely or partially in the early stages of the pandemic to reduce COVID-19 transmission. The institution took various actions using different social media platforms, mainstream media such as television channels, and distributing information via a free call center, the hospital continues to raise awareness of COVID-19 symptoms, prevention measures, and what to do if customers have symptoms or worries.

The hospital has devised a three-pronged strategy for combating COVID-19, including non-COVID services, an isolation facility for suspect patients, and a COVID-19 treatment center with its own personnel and restricted space for confirming patients. Different teams (sample team, ambulance team, Personal Protective Equipment (PPE) quantification team, infection prevention and control (IPC) team, safety & quality team specialized to COVID-19 isolation and treatment center) were organized in addition to the triple arrangement. This makes early testing and notification of suspect patient results easier and more convenient, as well as contact tracing, patient transfer, rational use and supply of PPE, and the availability of standard and transmission-based precaution facilities, all of which help to ensure that proper IPC measures were taken and maintained, as well as that the services delivered are of the expected quality. In addition to the aforementioned procedures, a task force unit made up of hospital executives and key individuals in charge of various service areas and units that directly or indirectly affect COVID-19 service was formed. This task force is in charge of overcoming obstacles, making crucial choices, and developing strategies.

When comparing the findings of this study to those of other similar studies, studies conducted in 22 French hospitals revealed a 26% reduction in all emergency department visits [9, 13]. In comparison to the pre-COVID era, this study found a 47% decrease in the percentage of patients attending all emergency departments of the hospital. COVID 19 occurrences had a significant relationship with a decrease in the number of patients visiting the emergency department (*P* > 0.0420) (Table 3).

**Table 3:** Chi -square test between per- COVID-19 and COVID-19 group

Essential healthcare type	per- COVID-19 season(average)	COVID-19 season (average)	***P*** value
**Out patient**			0.0126 ^*^
Internal Medicine	13,888	6489	
Surgery	6431	3000	
Gynaecology	3620	1105	
Dermatology	1268	289	
Dental & Maxillofacial	1351	324	
Ophthalmology	4603	1569	
Psychiatry	1460	647	
Ear Nose & Throat (ENT)	1651	555	
Renal Transplant	328	275	
Palliative care	5	3	
Oncology	1022	879	
Pediatrics	2112	1006	
**Emergency**			0.0420 ^*^
Pediatrics Emergency	554	164	
Adult Emergency	1526	485	
Gynaecology Emergency	1144	1028	
AaBET Emergency	966	527	
**Other services**			0.0657
Family planning	440	223	
Cervical cancer screening	16	6	
Voluntary Counselling & Testing	67	14	
Post Exposure Prophylaxis	10	3	
Dialysis	144	68	
**Maternal Service**			0.0947
Spontaneous Vaginal Delivery	505	462	
Instrumental delivery	60	37	
Caesarean section	368	376	
Maternal death	0	0	
Antenatal care	2084	1746	
Postnatal care	636	546	
**Surgical Service**			0.1623
Emergency surgery	254	193	
Elective surgery	384	168	
**Inpatient Service**			0.0638
Surgical ward	355	128	
Medical ward	84	49	
Pediatrics ward	68	49	
Maternity ward	497	354	
Gynaecology ward	154	91	
Others	886	504	
Total	2044	682	

* Significant association (*p*-value < 0.05)

The attendance for the three-month period from March to May 2020 (COVID period) in Ireland’s pediatric emergency department was 21,545. From 39,772 in the same period in 2018/2019, there was a 46% reduction [18]. This study in the pediatric emergency department yielded a substantially higher result. During the COVID period, attendance plummeted by 70% from 554 to 164 patients in average.

Our findings show that maternal services declined by 13% (3671 to 3177), which is similar to the Population Foundation of India’s projection of a 10% drop in coverage of pregnancy-related services [19]. However, when contrasted to the findings from the Ebola outbreak in 2014 and 2015, where facility-based deliveries plummeted by 28%, this study shows better maternal service attendance [10].

Maternal death was anticipated to rise by 8% in 2020 after the outbreak of this pandemic [14] by global finance professors, however our research found no increase in maternal mortality when compared to the same period previous to the outbreak.

In a study conducted in Switzerland, urgent operations were shown to have decreased by 39% (all specialties, 1476 vs. 897, *p* < 0.01) [15]. Our research yielded a more positive result. During the COVID-19 pandemic, there was a 24% decline in the number of emergency surgeries performed. It is evident that having a separate isolation surgical room and performing emergency procedures regardless of the patient’s COVID-19 result while maintaining the appropriate IPC precautions will result in this.

Regarding elective visceral surgeries, the study in Switzerland showed a 43% reduction after the emergence of COVID-19 (295 vs 165, *p* < 0.01) [15]. The higher result was found in this study, which showed a 56.3% (384 to 168) reduction in elective surgeries during the COVID-19 pandemic. This could be attributed to the fact that elective surgeries are performed after the RT-PCR test for COVID-19 is negative and the result arrives within 72 h of the surgery. The result usually takes 48–96 h. Outpatient surgical consultations dropped by 59%, from 728 to 296 (*p* < 0.01) in the Switzerland study [15]. Similar results were observed in this study. The number of patients visiting surgical OPD decreased by 53.35% (6431 to 3000 as a comparison between before and after the emergence of COVID-19.

When we compared the number of patients receiving essential health services six months after COVID-19 emerged, we found a gradual increase in all services except maternal services, which fell by 13%. (3104 to 2686). This could be owing to an influx of patients from other private and public healthcare institutions, hospitals, and homes during the early stages of the epidemic, as a result of the complete or partial shutdown of most facilities due to fear, stigma, and misinformation.

This study showed a significant association between the occurrence of COVID 19 and the number of clients seen in outpatient (*p* < 0.0126) and emergency departments (*p* < 0.0420) (Table 3). This may be due to extended appointments for chronic patients and restrictions on the number of patients seen in outpatient clinics. In the emergency department, it could be the fear of contracting COVID 19 while visiting for other concerns.

## Conclusion

The number of clients visiting almost all essential health care services declined during the COVID-19 pandemic. The most affected services were inpatient admissions that showed a 73% (2044 to 682) reduction from the previous year and the list of affected maternal services only decreased by 13% (3671 to 3177). During six months of follow up after the emergence of COVID-19, a progressive increment in the number of patients getting essential services was observed.

Essential healthcare services are as important as COVID-19 prevention and treatment. These services should be provided alongside COVID-19 services. We can improve the number of clients visiting hospitals in times of a pandemic by endorsing a continuous information campaign with available media, implementation and monitoring of IPC measures, and establishing a holistic triple setup for delivering quality medical service in times of a pandemic.

## Recommendation

1. At the national level the health care system should be rearrangement the classification of “COVID” and “non-COVID” health facilities in each town in the event of a particularly significant caseload. This helps us to limit the danger of contamination, enhance patient routes, and combine health facilities and skilled caregivers; while on the other hand, it allows us to continue managing non-COVID patients without raising risk.

2. At national level there should be continues awareness creation of COVID-19 symptoms, prevention measures and treatment options. Develop educational materials and disseminate using different social media platforms and mainstream media such as television channels.

3. At hospital level we recommend creating a triple setup to battle COVID-19, which includes non-COVID services, an isolation center for suspect patients, and a COVID-19 treatment center with its own personnel and restricted access.

### Limitation

We did not include all essential services and it was conducted in a single hospital.

## Data Availability

The datasets used and/or analysed during the current study are available from the corresponding author upon reasonable request.

## Abbreviations

COVID-19: Coronavirus disease
SPHMMC: St. Paul’s Hospital Millennium Medical College
SPSS: Statistical Package for Social Sciences
PPE: Personal Protective Equipment
WHO: World Health Organization
